# Oncolytic adenovirus encoding LHPP exerts potent antitumor effect in lung cancer

**DOI:** 10.1038/s41598-024-63325-z

**Published:** 2024-06-07

**Authors:** Yaru Zhao, Huihui Liu, Qi Zhan, Hao Jin, Yiqiang Wang, Hui Wang, Biao Huang, Fang Huang, Xiaoyuan Jia, Yigang Wang, Xiaoyan Wang

**Affiliations:** 1 College of Life Sciences and Medicine, Zhejiang Sci-Tech University, Hangzhou, 310018 China; 2 Oncology Department, Zhejiang Xiaoshan HospitaI, Hangzhou, China; 3 Surgical Department of Duchang County Second People’s Hospital, Jiujiang, 332600 China; 4 Department of Pathology, Zhejiang Provincial People’s Hospital, Hangzhou, 310014 China

**Keywords:** Cancer, Biological techniques

## Abstract

LHPP has been shown to be a new tumor suppressor, and has a tendency to be under-expressed in a variety of cancers. Oncolytic virotheray is a promising therapeutics for lung cancer in recent decade years. Here we successfully constructed a new recombinant oncolytic adenovirus GD55-LHPP and investigated the effect of GD55-LHPP on the growth of lung cancer cells in vitro and in vivo. The results showed that LHPP had lower expression in either lung cancer cells or clinical lung cancer tissues compared with normal cells or tissues, and GD55-LHPP effectively mediated LHPP expression in lung cancer cells. GD55-LHPP could effectively inhibit the proliferation of lung cancer cell lines and rarely affected normal cell growth. Mechanically, the oncolytic adenovirus GD55-LHPP was able to induce stronger apoptosis of lung cancer cells compared with GD55 through the activation of caspase signal pathway. Notably, GD55-LHPP also activated autophagy-related signal pathway. Further, GD55-LHPP efficiently inhibited tumor growth in lung cancer xenograft in mice and prolonged animal survival rate compared with the control GD55 or PBS. In conclusion, the novel construct GD55-LHPP provides a valuable strategy for lung cancer-targeted therapy and develop the role of tumor suppress gene LHPP in lung cancer gene therapy.

## Introduction

According to the latest statistics, the incidence and mortality rates of lung cancer rank first in all cancer types throughout the worldwide, and the 5 year overall survival rate is less than 20%^1,2^.Current treatment strategies such as surgery, chemotherapy, radiotherapy, and molecular targeting therapy as well as immunotherapy are ineffective against advanced tumors. Therefore, there is still an urgent need to search novel approaches to treat lung cancer.

Oncolytic virotherapy has recently been considered as one of the most promising strategies for the treatment of solid tumors due to its tumor-targeting ability and high efficiency^3,4^. Our group has been working on Cancer-targeting gene-virotherapy (CTGVT) through inserting anti-tumour genes into oncolytic virus vectors, such as adenoviral vector and vaccinia vector^5,6^, which has obtained better anti-tumour effect compared to oncolytic virus alone and gene therapy alone.

GP73, is a Golgi glycoprotein, also called GOLPH2 and is upregulated in a wide range of cancers^7,8^. It is an excellent candidate marker for hepatocellular carcinoma, and its specificity is even better than that of the hepatocellular carcinoma marker AFP^9^. Previously, we constructed a GP73-regulated oncolytic adenovirus (GD55) based on ZD55 of CTGVT, exhibits a stronger oncolytic effect compared to common oncolytic adenovirus in human liver cancer stem-like cells^10^, human prostate cancer stem-like cells^5^, and hepatocellular carcinoma^11^either in vivo or in vitro. It showed that GD55 has an excellent antitumor potential as oncolytic virus vector to deliver tumor suppression gene.

In recent years, phosphoinositide phosphohistidine inorganic pyrophosphate phosphatase (LHPP), a histidine phosphatase, has been shown to be a new tumor suppressor^12^.Low expression of LHPP has also been found in cancer tissues of bladder cancer^13^, lung cancer, pancreatic cancer^14,15^, cervical cancer^16^, papillary thyroid cancer^17^, and colorectal cancer^18,19^. Overexpression of LHPP protein inhibits hepatocellular carcinoma cell growth and metastasis, and also reduces the transcription of CCM1, PKM2, MMP7, and MMP9 in cancer cells, and that high expression of LHPP was negatively correlated with cell cycle and metastasis, among others^20^. It suggests that LHPP is lung cancer suppressor with potent.

In this study, we constructed a novel oncolytic adenovirus GD55-LHPP in which GD55 was used to carry LHPP gene, and investigated the role of GD55-LHPP on lung cancer cells. The results showed that GD55-LHPP mediated high expression of LHPP in lung cancer cells and inhibited efficiently lung cancer cell growth in vivo and in vitro. Thus, our results implied that GD55-LHPP might act as a promising therapeutic agent for lung cancer.

## Result

### Low expression of LHPP in lung cancer cells and tissues

**Figure 1:**
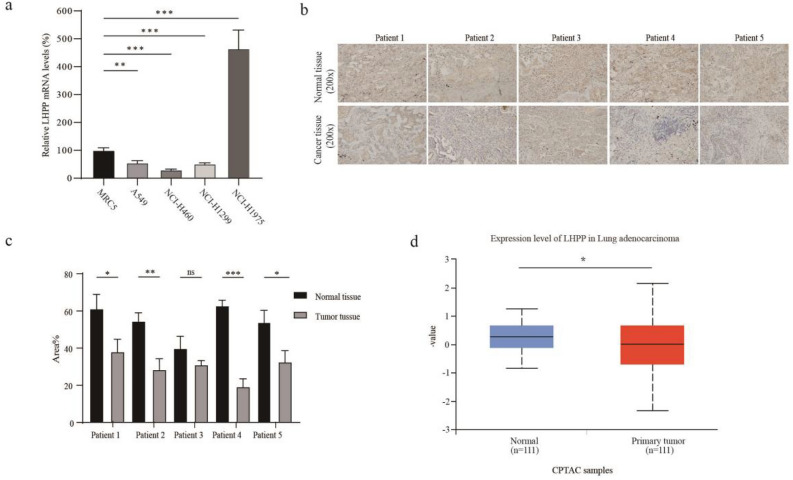
Low expression of LHPP in lung cancer cells and tissues (**a**) The expression of LHPP mRNA in normal lung cell lines and different lung cancer cell lines by RT-qPCR . **P < 0.01, ***P < 0.001. (**b**) LHPP expression in five lung cancer tissue specimens and paracancer normal tissues (Orginal amplification: × 200). (**c**) The quantitative analysis of the LHPP expression staining images in five lung cancer tissue specimens and paracancer normal tissues. **P < 0.01, ***P < 0.001. (**d**) The expression of LHPP in lung adenocarcinoma derived from CPTAC samples.

The expression of the tumor suppressor LHPP is often absent or lowered in various types of human cancers due to the loss of heterozygosity and promoter hypermethylation^21^. Here, we first detected LHPP expression in several lung cancer cell lines, and found LHPP expression was down-regulated in detected lung cancer cells except H1975 compared with the human embryonic lung cell line MRC-5 in mRNA level. (Fig. 1a, Supplementray Table 1). Furthermore, it was found that LHPP expression was clearly weakened in five lung cancer tissue specimens compared with paracancer normal tissues (Fig. 1b and c). It is consistent with low expression of LHPP in lung adenocarcinoma derived from CPTAC samples (https://ualcan.path.uab.edu/) (Fig. 1d). The above results suggest LHPP might be a candidate lung cancer suppressor.

### Construction of recombination oncolytic virus GD55-LHPP and its characteristic

**Figure 2:**
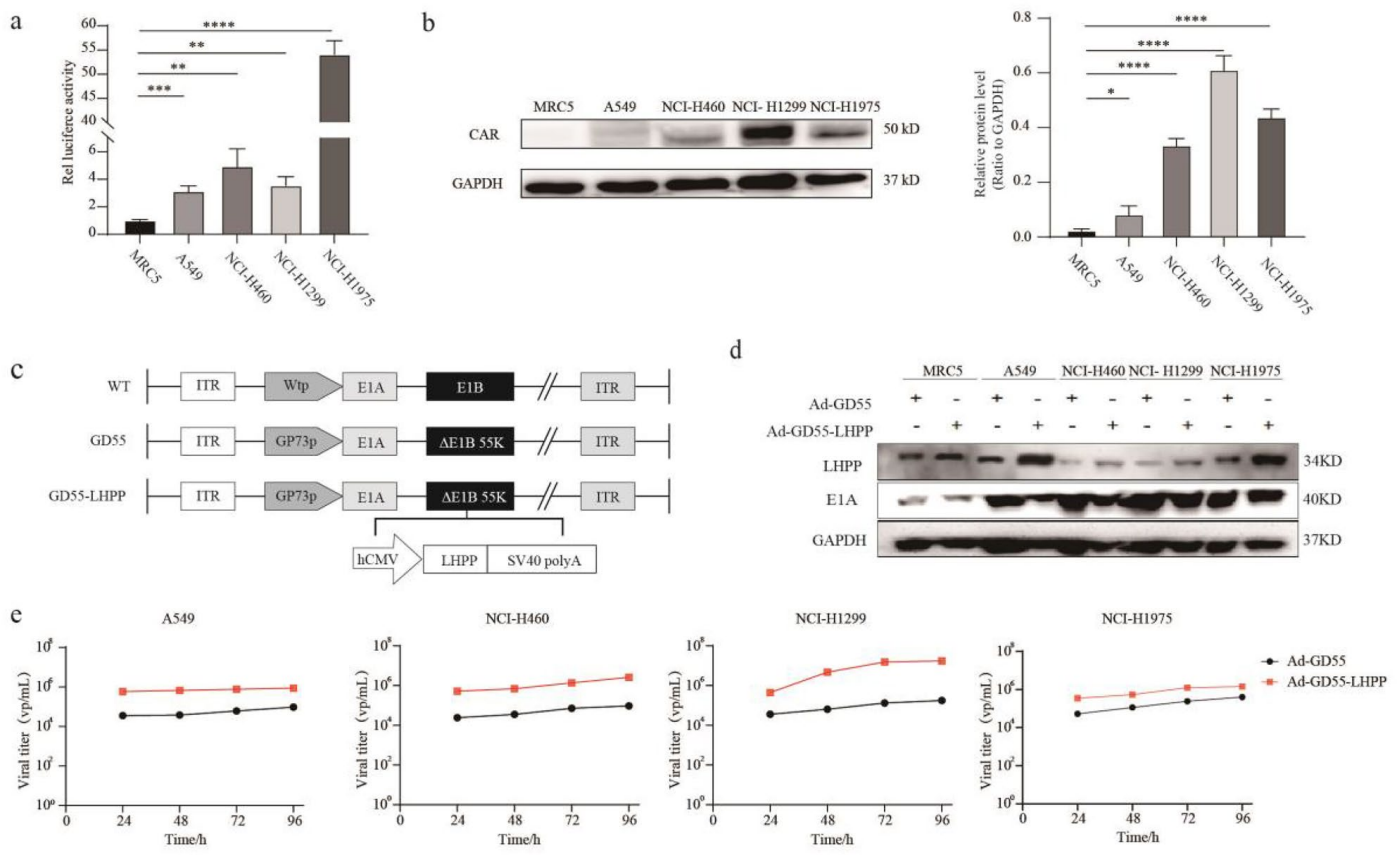
Construction of recombination oncolytic virus GD55-LHPP and its characteristic. (**a**) The promoter activity of the GP73 promoter in normal lung cell lines and different lung cancer cell lines **P < 0.01, ***P < 0.001. (**b**) The expression of Coxsackie and Adenovirus Receptor (CAR) protein in normal lung cell lines and different lung cancer cell lines by Western blotting. **P < 0.01, ***P < 0.001. (**c**) Schematic diagram of the structure of the recombinant oncolytic adenovirus structure. ITR, inverted terminal repeat. (**d**) The expression of LHPP protein in four lung cancer cell lines (A549, H460, H1299, and H1975) and one human embryonic lung cell (MRC-5) infected with GD55-LHPP or GD55 at the 10 MOI for 48 h by Western blotting. (**e**) The progeny replication of the virus replicates in four lung cancer cell lines (A549, H460, H1299, and H1975) were infected with 10 MOI of GD55-LHPP or GD55 for 24, 48, 72, 96 h.

We have previously successfully constructed an oncolytic adenovirus GD55 under the control of GP73 promoter^7^. GP73 is a potential tumour biomarker, especially in lung, liver, and prostate cancers. Herein, we examined the promoter activity of the GP73 promoter in different cell lines and showed higher promoter activity in four lung cancer cell lines compared with lung normal cells (Fig. 2a). In order to verify whether the higher expression of Coxsackie and Adenovirus Receptor (CAR) and specific for adenoviral infection in lung cancer cells than in normal cells, we examined the expression of CAR in four types of lung cancer cells and normal cells and found that CAR was highly expressed in lung cancer cells (Fig. 2b). Subsequently, we generated a novel oncolytic adenovirus GD55-LHPP by inserting the LHPP gene into E1B 55 K deleting locus (Fig. 2c). Then, we evaluated expression of LHPP and E1A mediated by GD55 as well as progeny replication ability of GD55-LHPP in lung cancer cell lines. The result indicated that the high expression of LHPP gene was observed in GD55-LHPP-infected A549, H460, H1299 and H1975 cells than that of GD55, and whether GD55-LHPP or GD55 can result in adenovirus E1A expression in the above lung cancer cells compared with the normal MRC-5 cells (Fig. 2d). The progeny replication assay indicated that GD55-LHPP exerts higher progeny virus multiplication ability with GD55, implying that insertion of LHPP gene might have the potential to increase the replication of the viral progeny(Fig. 2e).

### Killing effect of GD55-LHPP on lung cancer cell lines

**Figure 3:**
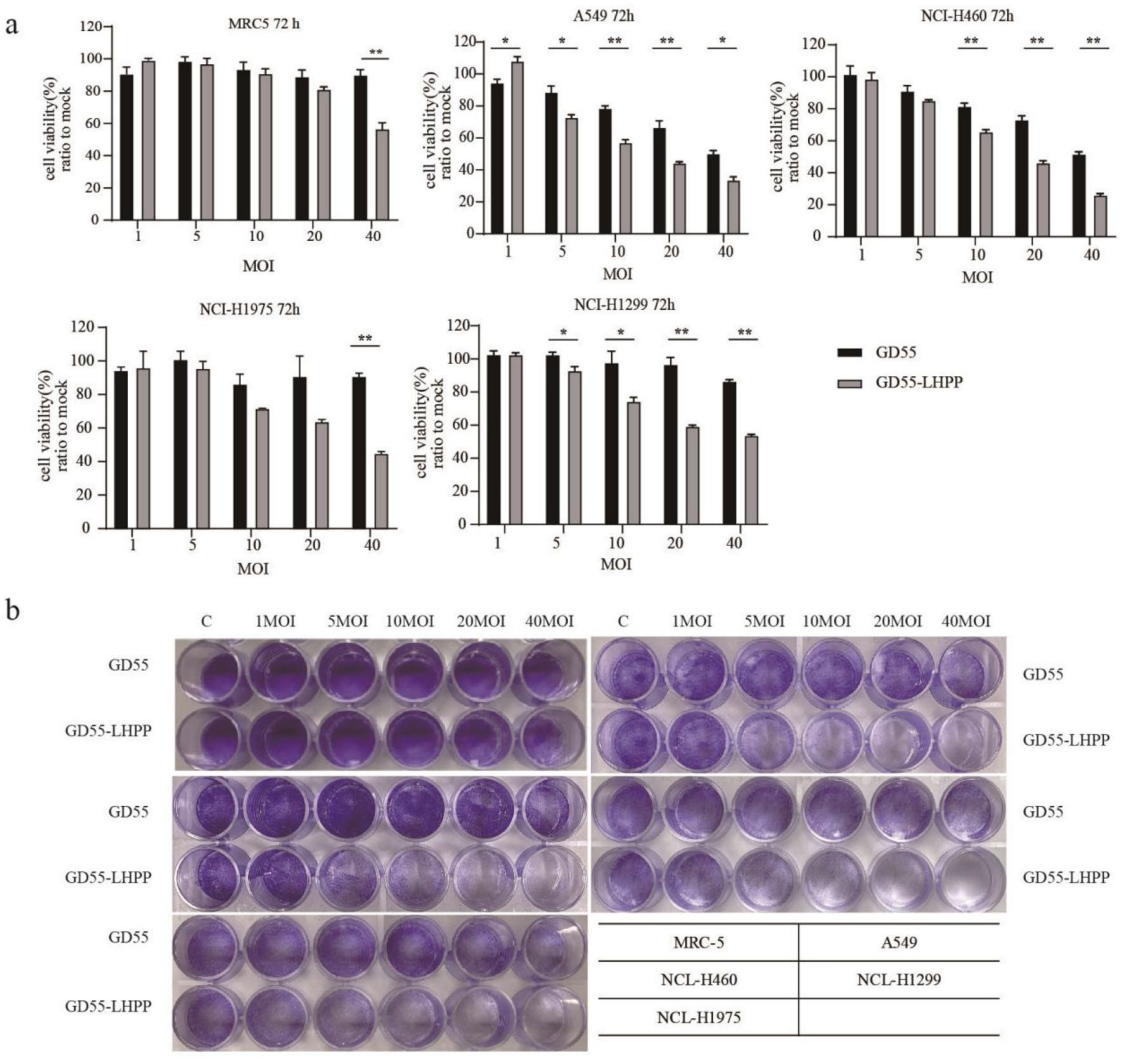
Killing effect of GD55-LHPP on lung cancer cell lines. (**a**) MTT assay was used to detect the anti-tumor effect of GD55 and GD55-LHPP in one human embryonic lung cell MRC-5 and lung cancer cells NCI-H460, A549, NCI-H1299, NCI-H1975. *P < 0.05, **P < 0.01, ***P < 0.001 (**b**) Crystal violet assay was employed to determine the cytopathic effect of GD55 and GD55-LHPP in one human embryonic lung cell (MRC-5) and four lung cancer cells (A549, H460, H1299, and H1975). MOI, the multiplicity of infection.

To evaluate the anti-tumor effect of GD55-LHPP in vitro, four lung cancer cell lines (A549, H460, H1299, and H1975) and one human embryonic lung cell (MRC-5), were infected with various concentrations of GD55-LHPP for 72 h. The MTT assay indicated that GD55-LHPP had a greater inhibitory effect than GD55 (Fig. 3a). The crystal violet assay revealed that GD55-LHPP provoked a greater cytopathic effect on lung cancer cells than GD55(Fig. 3b). In addition, it is noted that whether GD55-LHPP or GD55 showed slight suppression effect to normal cells, suggesting recombinantion oncolytic virus has good targeting and safety.

### GD55-LHPP induced cell apoptosis and its mechanism in lung cancer cells

**Figure 4:**
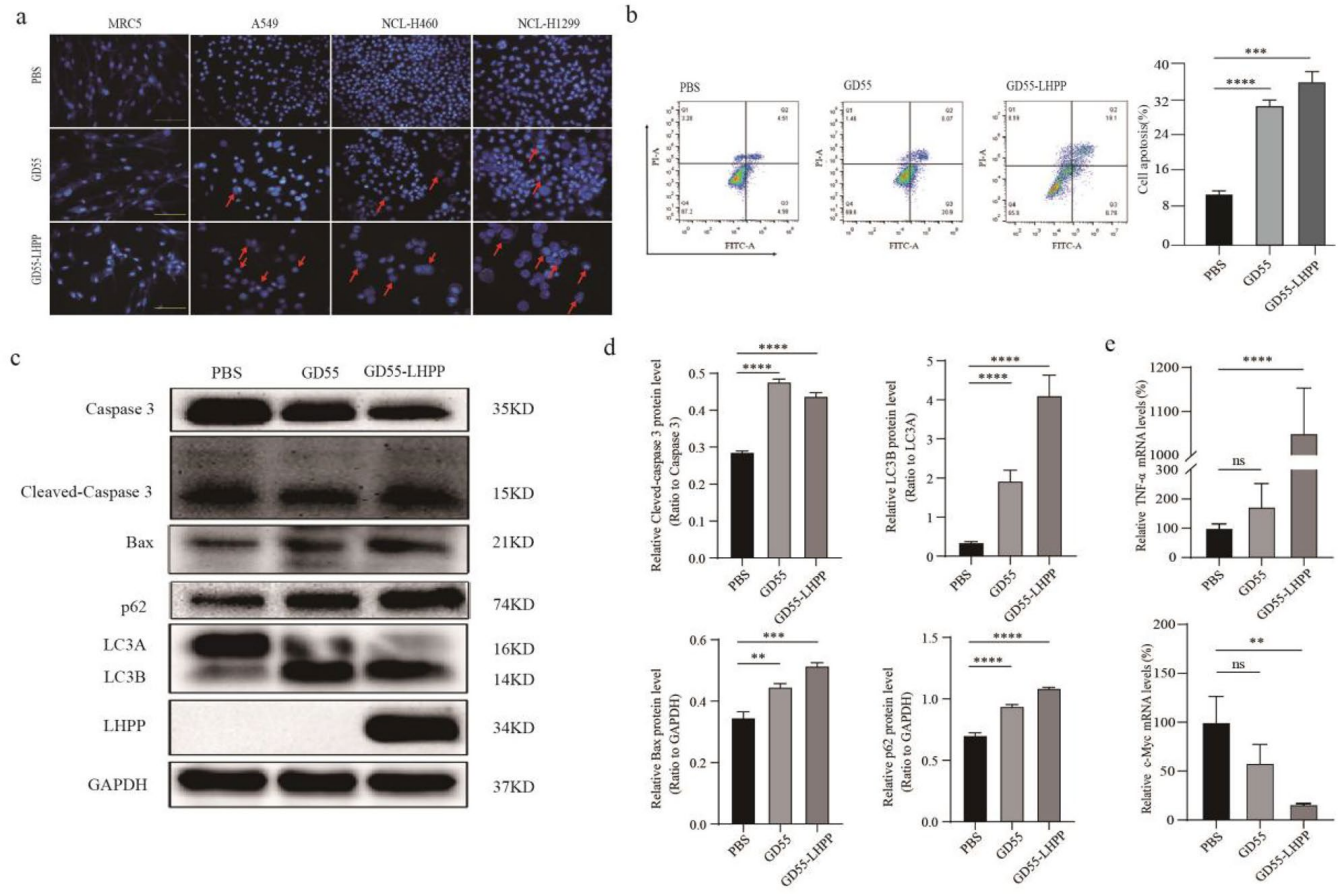
GD55-LHPP induced cell apoptosis and its mechanism in lung cancer cells. (**a**) After 48 h, nuclear fragmentation (arrows) was observed in MRC-5, A549, NCI-H460, NCI-H1299 cells (0.2 mm fields; original magnification, × 200) in different treatment groups using Hoechst staining under an inverted fluorescence microscope. (**b**) The percentage of apoptotic cells in NCI-H460 cells treated with PBS, GD55, or GD55-LHPP was detected using flow cytometric analysis. The data are presented as the mean ± standard deviation. *P < 0.05, **P < 0.01, ***P < 0.001. (**c**) and (**d**) Expression of apoptosis-associated proteins expression was detected by Western blotting in NCI-H460 after treatment with PBS, GD55, or GD55-LHPP. GAPDH was used as the internal control. (**e**) Expression of TNF-α, and c-Myc mRNAs in NCI-H460 cells treated with PBS, GD55, or GD55-LHPP. *P < 0.05, **P < 0.01, ***P < 0.001.

To detect whether the inhibition of cell proliferation by GD55-LHPP was due to the activation of apoptosis, this form of cell death was analyzed. The apoptotic morphological analysis by Hoechst 33342 staining showed that GD55-LHPP triggered a higher level of nuclear fragmentation, the formation of apoptotic bodies, and chromatin condensation, inducing a higher magnitude of cell apoptosis than GD55 (Fig. 4a). Additionally, flow cytometric analysis documented that the rate of cell apoptosis in the GD55-LHPP group was markedly increased compared with the NC group or the GD55 group in NCI-H460 (Fig. 4b).

To identify the mechanism of apoptosis activation, the caspase signaling pathway was analyzed in NCI-H460. The result indicated that GD55-LHPP significantly increased the expression of LHPP, Cleaved-Caspase 3, and pro-apoptotic protein Bax, as well as autophagic marker protein p62, and converted more LC3A proteins to LC3B proteins (Fig. 4c and d). Meanwhile GD55-LHPP significantly increased the expression of TNF-α, and demoted the expression of c-Myc (Fig. 4e, Supplementray Table 2). The results showed that recombinant oncolytic adenovirus GD55-LHPP induces tumour cell death by activating the intracellular caspase-related apoptosis and autophagic signaling pathway.

### Antitumor effect of GD55-LHPP in mice

**Figure 5:**
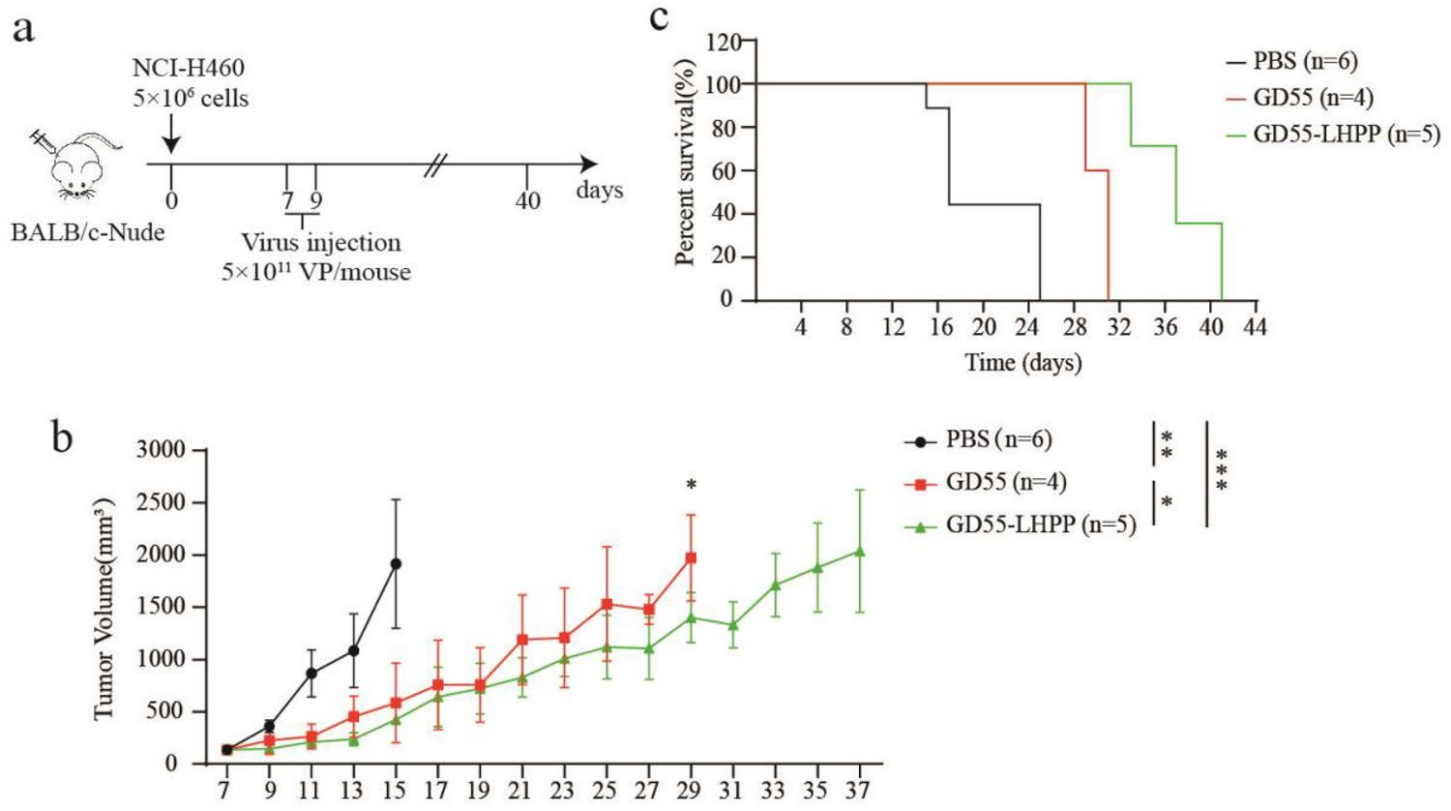
Antitumor effect of GD55-LHPP in mice. (**a**) A schematic diagram of building a tumor xenograft model and injecting an oncolytic virus. (**b**) The tumour volume was measured every 2 days using the formula V (mm3) = 0.52 × length × width2. Data are presented as the mean ± standard deviation, n = 5. *P < 0.05, **P < 0.01, ***P < 0.001. (**c**) The mouse survival rate in different treatment groups.

**Figure 6:**
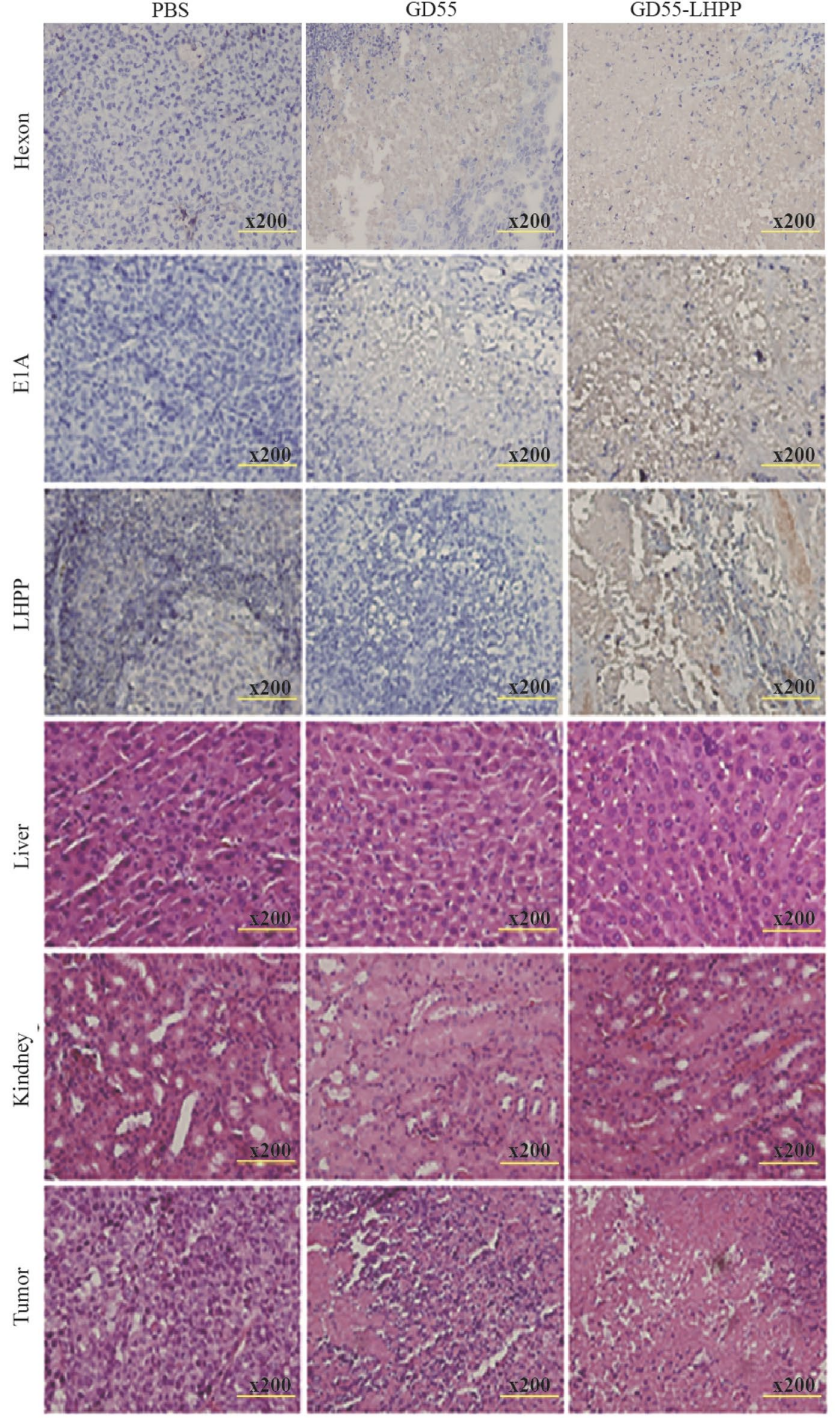
Histopathology assay. IHC and HE staining for detection of oncolytic adenovirus amplification and hepatorenal toxicity in different treatment groups. Magnification, × 200.

To evaluate the antitumor efficacy of intratumoral administration of GD55-LHPP in nude mice carrying human lung cancer xenografts. Based on in vitro cell cytotoxicity results, GD55-LHPP exhibited most effective inhibitory effect in NCI-H460 cells compared with other lung cancer cells, thus NCI-H460 cells was used as further in vivo study. Subsequently, NCI-H460 cells were subcutaneously implanted into nude mice at 5 × 10^6^ cells; a single dose of GD55-LHPP or control is injected intratumorally at 5 × 10^11^ VP when the tumor volume reached about 80–120 mm^3^ and a second injection is given two days apart (Fig. 5a). The result shows that compared with the PBS treatment group, the tumor growth rate of nude mice in the virus treatment group was significantly reduced, and the survival time of mice was significantly prolonged. Especially, all mice in the PBS group died after 25 days of treatment and the tumor volume in the GD55-LHPP-treated group was only 1 400.75 mm^3^ compared that of 1 974.19 mm^3^ in the GD55-treated group at 29 days (Fig. 5b and c). The results of immunohistochemistry showed that the recombinant oncolytic virus efficiently mediated expression of virus Hexon and E1A protein, and GD55-LHPP mediated obvious LHPP expression at the tumor site (Fig. 6 upper). HE staining showed that our oncolytic virus can induce cell death in tumor tissues, and had no obvious toxic effect on liver and kidney tissues of mice (Fig. 6 lower). It suggests our constructed oncolytic virus can efficiently replicate and induce cell death in tumor tissues with good safety.

## Discussion

As an emerging cancer therapeutic strategy, oncolytic virus (OV) therapy has been attracted more and more attention because of its good achievements in preclinical and clinical studies^22^. OVs are able to target proliferate in tumour cells and ultimately lyse them without harming normal tissues, and simultaneously trigger the host’s innate and adaptive antitumor immune response^23–25^. After the tumour cells are lysed, the viruses within them are also released to infect and kill other tumour cells^26,27^, which forms a cascade amplification effect. In recent years, several types of OVs have been approved, such as H101^28^ in China, T-VEC^29^ in the USA, and the Japanese herpes simplex virus Deltyact^30^. These marketed OV drugs bring a good prospect and important motivation for the development of OVs. However, the tumour-killing effect of OVs is still insufficient in current research, thus the modification of OVs and delivery of novel tumour suppressor to improve their safety and antitumor effect is a major trend for future research.

Among all used OV vectors, adenovirus (Ad) is the most used viral vector that can efficiently infect almost all types of tumor cells. There are some drawbacks for adenovirus to design as an OV drug, such as the preexisting neutralization antibodies of adenovirus in the human body prone to clear virus and lack of tumor targeting. Thus it is needed to modify adenovirus to overcome these obstacles. To improve tumor-targeting of oncolytic Ad, our group has put forward to the CTGVT strategy and designed an independent intellectual property rights of oncolytic adenovirus ZD55 by deleting Ad E1B 55 kDa gene to target p53-deficient tumor cells^10^. To further improve transcriptional targeting ability of ZD55, we previously constructed a GP73-regulated OV GD55, and demonstrated that GD55 confers high adenovirus replication, infectivity and antitumor effect^9,10^. Therefore, recombinant oncolytic adenovirus GD55 has a promising anti-tumor potential especially through delivering novel tumour suppressor genes.

LHPP is a histidine phosphatase, and recently LHPP has been identified to be a tumour suppressor in HCC and other cancers^31,32^, which can inhibit tumour cell proliferation and metastasis, and induce apoptosis in numerous tumor cells by affecting different target genes^33^. Mechanically, tumor suppression activity of LHPP may be mediated through inhibiting EGFR^34^, modulating AKT/mTOR^35,36^ and TGF-β/smad signaling pathway^18^ etc. Recent study also proved that loss of LHPP is associated with intestinal inflammation in colitis mice model and inflammatory bowel disease patients^21^, showing that vital roles of LHPP in maintenance of normal body functions. Because histidine phosphorylation belongs to a hidden phosphoproteome and is a poorly understood and uncharacterized post-translational modification of proteins^31^, the detailed functions of LHPP as a histidine phosphatase still need to be classified. To be noted, GD55-LHPP showed a certain high progeny replication potential compared with GD55, although some tumour cells showed significant differences, others did not (Fig. 2C). The possible reason is that the oncolytic virus GD55 used here lacks E1B55 kDa gene, but retains the early gene E4orf1. However, E4orf1 restricts the expression of late viral proteins of E1B55 kDa-missing oncolytic adenoviruses such as ONYX-015, thereby reducing progeny viral replication^37^. The mechanism is that E4orf1 induces the activation of PI3K/Akt signal, leading to phosphorylation of Akt^37^. Since LHPP could inhibit the Akt signaling pathway, thus GD55-LHPP overexpressing LHPP has the potential to increase viral progeny replication. Moreover, although we have preliminarily found that GD55-LHPP can inhibit the proliferation of tumor cells, the specific mechanism of action and the effect on normal cells have not been clearly classified in this study. Thus, further studies are needed to confirm the roles of LHPP.

Herein, the anti-tumour effect of GD55-LHPP was demonstrated in lung cancer cells and the NCI-H460 xenograft mouse model, which was consistent with our expected results.

However, there are still some shortcomings to be improved in further study. In Fig. 3a, the cell viability was reduced in MRC-5 normal lung cells infected with 40 MOI of GD55-LHPP, implying that its toxicity in normal cells. The possible reason is the effect of LHPP-overexpression mediated by high viral dose in normal cells. Thus, the safety of our constructed oncolytic adenovirus in the clinical application needs to be further evaluated. For example, and further modification of oncolytic adenovirus can enhance its safety in clinical application^38–40^. Meanwhile, its anti-tumour efficacy can also be enhanced by combining with other therapeutic modalities. Studies have shown that combining oncolytic viruses with chemotherapeutic agents^41^, CAR-T^42,43^ or immune checkpoint inhibitor molecules, such as CTLA4^44,45^、PD-1/PD-L1^44^、Tim3^45^、Siglec-5^46^, significantly increased their anti-tumour efficacy.

In conclusion, our study confirmed that recombinant oncolytic virus GD55-LHPP could inhibit cell proliferation both in vivo and in vitro in lung cancer cells, and induce tumor cells death by activating the caspase signaling pathway, as well as autophagic pathway. Therefore, this study confirmed the role of LHPP gene and the broad prospect of oncolytic virus GD55-LHPP for lung cancer treatment.

## Materials and methods

### Real-time quantitative polymerase chain reaction (RT-qPCR)

The treated cells were collected and washed twice with pre-cooled PBS; secondly, each sample was lysed by adding 1 mL of Trizol on ice for 3 min; finally, the lysate was collected into 1.5 mL RNase free EP tubes for mRNA extraction. The extracted mRNA was reverse transcribed by reverse transcription kit to obtain cDNA (Cowin Biotech, China), and the experiments were performed according to the instructions of SYBR Green (Vazyme Biotech, China) in 7500 Fast Real-Time PCR System. The primers used in the experiment were:

- LHPP qRT-PCR F: 5ʹ-CTGTGTGGTAATTGCAGACGC-3ʹ
- LHPP qRT-PCR R: 5ʹ-TAGTAACGCCCTTTTCCCAGT-3ʹ
- GAPDH qRT-PCR F: 5ʹ-ACAACTTTGGTATCGTGGAAGG-3ʹ
- GAPDH qRT-PCR R: 5ʹ-GCCATCACGCCACAGTTTC-3ʹ
- c-myc qRT-PCR F: 5ʹ-ACAGCGTCTGCTCCACCT-3ʹ
- c-myc qRT-PCR R: 5ʹ-ATCCAGCCGCCCACTTTT-3ʹ
- TNF-α F: 5ʹ-GAGGCCAAGCCCTGGTATG-3ʹ
- TNF-α R: 5ʹ-CGGGCCGATTGATCTCAGC-3ʹ

### Ethics approval

Lung cancer samples were obtained from Zhejiang Xiaoshan Hospital and performed in accordance with relevant guidelines and regulations. The study was approved by the Ethics Committee of Zhejiang Xiaoshan Hospital. Written informed consent was received from all participants prior to inclusion in the study. Animal studies were approved by the Laboratory Animal Ethics Committee of Zhejiang Sci-Tech University (ZSTU) and performed in accordance with the guidelines and regulations of the ZSTU.

### Cell lines and culture

The human embryonic lung cell line MRC-5, human lung adenocarcinoma cell lines A549, NCI-H460, NCI-H1299, NCI-H1975 and human embryonic kidney cell lines HEK293A, HEK293T were obtained from Shanghai Institute of Cell, Chinese Academy of Sciences. All cells were cultured in Dulbecco’s modified Eagle’s medium (DMEM) with 10% FBS, 100 U/mL of penicillin, and 100 mg/mL of streptomycin in a 5% CO_2_, 37 °C incubator.

### Western blot analysis

Western blot analysis was performed according to previous description. Briefly, the cells were harvested and lysed, and protein concentration was determined with Pierce BCA protein assay kit (Thermo Fisher Scientific). Cell proteins were subjected to SDS–polyacrylamide gel electrophoresis. Separated proteins were transferred onto polyvinylidene difluoride membranes and incubated with primary antibodies against procaspase-3, cleaved caspase-3, Bax, p62, LC3A, and LC3B (Cell Signaling Technology, Danvers, MA, USA), LHPP, CAR, GAPDH, and Beclin1 (Santa Cruz Biotechnology, Santa Cruz, CA, USA).

### Dual luciferase reporter assay

The luciferase reporter assay for various GP73 promoter truncations was done according to the Dual-glo luciferase assay kit (GeneCopoela, LF002). Briefly, cells were seeded in 6-well culture plate overnight before transfection. The pGL3-L659 (p-677/-19) (which was constructed by previous study^9^) and the pRL were cotransfected into the lung cancer cell lines A549, NCI-H460, NCI-H1299, NCI-H1975 and lung embryonic fibroblasts MRC-5 at a ratio of 1:50 to test the GP73 promoter activity of GP73 promoter in different cell lines.

### Crystal Violet staining

The cells were seeded in 24-well plates (3 ~ 5 × 10^4^ cells/well), treated with GD55-LHPP and GD55 at indicated MOIs (1, 5, 10, 20, and 40) for 72 h, and stained with 0.5% crystal violet solution. Subsequently, the cells were gently washed with PBS twice and dried at 37 ℃ and photographed. Uninfected cells served as a control.

### Cell viability assay

The cells were inoculated into 96-well plates at 3 ~ 5 × 10^3^ cells/well, and treated with GD55-LHPP and GD55 via various multiplicity of infection (MOI: 1, 5, 10, 20, 40) after the cells are attached to the well. After 72 h, cell viability was examined by methyl thiazolyl tetrazolium (MTT; Sigma-Aldrich, St. Louis, MO, USA) assay. Briefly, 20 µL MTT (5 mg/mL in PBS) was added into each well, and after 4 h of incubation at 37 ℃, the mediums and MTT were removed, and 150 µL of Dimethyl Sulfoxide was added into each well. The plates were mixed thoroughly for 10 min, and cell viability was assessed by measuring the absorbance at 490 nm using a microplate reader. Each experiment was repeated three times.

### Hoechst 33342 assay

The cells were inoculated into 24-well plates (3 × 10^4^ cells/well). After the cells are attached and treated with GD55 and GD55-LHPP at the indicated MOI for 72 h. Subsequently, the cells were incubated in Hoechst 33342 solution (Beyotime, Shanghai, China) for 10 min and then washed twice with PBS. The cells were then observed under the IX71-22FL/PH fluorescence microscope (Olympus Corporation, Japan) at 200 × magnification.

### Flow cytometric analysis

Cell apoptosis was determined using Annexin V FITC/PI double staining kit (Biosciences, San Jose, CA, USA) according to our previous description^47^. Briefly, 5 × 10^5^ cells were inoculated into each well of 6-well plates, cultured for 12 h, and then treated with oncolytic adenovirus GD55-LHPP and GD55 for an additional 48 h. Subsequently, the cells were harvested by trypsinization without EDTA, resuspended in 500 µL of binding buffer, and stained with FITC-labeled annexin V and PI. The staining was immediately evaluated using the flow cytometer (BD Biosciences).

### Animal experiments

Animal experiments were performed according to the regulations and standards set by ARRIVE guidelines and were performed according to previous description^14^. The study was approved by the Laboratory Animal Welfare Ethics Committee of the Zhejiang Sci-Tech University (No. 20220105-05) in January 2022. Briefly, 4 weeks old female BALB/c nude mice were purchased from the Shanghai Experimental Animal Center. A total of 5 × 10^6^ NCI-H460 cells/mice were subcutaneously inoculated into the right flank of the mice. When the xenograft tumors reached 80–120 mm^3^, the mice were divided randomly into 3 groups (PBS, GD55, and GD55-LHPP). Each mouse was injected twice with PBS (vehicle), GD55 (5 × 10^8^ PFU), and GD55-LHPP (5 × 10^8^ PFU) every third day. After treatment, tumor size was measured with a vernier caliper every two days. Mice were euthanized when tumor volume was more than 2000 mm^3^. Tumor volume (V) was calculated according to the formula V (mm^3^) = 0.5 × length (mm) × width (mm^2^). Then, tumor, liver and kidney tissues were harvested, fixed in 5% paraformaldehyde, dehydrated in a gradient of increasing ethanol concentrations, and embedded in paraffin. 5 µm sections were cut and stained with hematoxylin and eosin for histological analysis. Adenovirus E1A and LHPP expression in tumor tissues were detected with immunohistochemical staining to valuate virus replication.

### Histopathology assay

For analysis of histopathology, mice have been randomly selected from each group and were killed after 20 days of the first treatment in different groups. Tumour tissue, heart, liver, kidney and spleen were harvested and fixed in 5% paraformaldehyde, dehydrated with gradient increasing ethanol concentrations and embedded in paraffin wax, which were cut in 5 µm sections. The sections were stained with haematoxylin and eosin for histological analysis. The sections were incubated with anti-E1A and anti-LHPP antibodies and then incubated with the avidin–biotin-peroxidase complex reagent (Vector Laboratories, Burlingame, CA, USA) for the IHC analysis. Haematoxylin was used as a counterstain.

### Statistical analysis

All data are presented as the mean ± standard deviation (SD). The differences were assessed by Student’s t-test and the analysis of variance (ANOVA) with GraphPad Prism 6 (GraphPad Software, Inc, CA, USA). p < 0.05 were statistically considered significant.

### Supplementary Information

Supplementary Tables.

Supplementary Information.

## Data Availability

All data generated or analysed during this study are included in this published article.

## References

[1] Siegel RL (2023). Cancer statistics, 2023. CA Cancer J Clin..

[2] Bade BC, Dela Cruz CS (2020). Lung cancer 2020: Epidemiology, etiology, and prevention. Clin. Chest Med..

[3] DePeaux K, Delgoffe GM (2023). Integrating innate and adaptive immunity in oncolytic virus therapy. Trends Cancer.

[4] Seyed-Khorrami SM, Azadi A, Rastegarvand N, Habibian A, Soleimanjahi H, Łos MJ (2023). A promising future in cancer immunotherapy: Oncolytic viruses. Eur. J. Pharmacol..

[5] Xiao B (2020). Oncolytic adenovirus CD55-Smad4 suppresses cell proliferation, metastasis, and tumor stemness in colorectal cancer by regulating Wnt/β-catenin signaling pathway. Biomedicines.

[6] Zhao L (2005). Potent antitumor activity of oncolytic adenovirus expressing mda-7/IL-24 for colorectal cancer. Hum. Gene Ther..

[7] Hu X (2023). Golgi-protein 73 facilitates vimentin polymerization in hepatocellular carcinoma. Int. J. Biol. Sci..

[8] Ying C (2018). GOLPH2-regulated oncolytic adenovirus, GD55, exerts strong killing effect on human prostate cancer stem-like cells in vitro and in vivo. Acta Pharmacol. Sin..

[9] Wang Y (2015). A novel Golgi protein (GOLPH2)-regulated oncolytic adenovirus exhibits potent antitumor efficacy in hepatocellular carcinoma. Oncotarget.

[10] Zhang X (2016). GP73-regulated oncolytic adenoviruses possess potent killing effect on human liver cancer stem-like cells. Oncotarget.

[11] Zhu H (2023). LHPP suppresses proliferation, migration, and invasion in hepatocellular carcinoma and pancreatic cancer by inhibiting EGFR signaling pathway. Med. Oncol..

[12] Li Y, Zhang X, Zhou X, Zhang X. LHPP suppresses bladder cancer cell proliferation and growth via inactivating AKT/p65 signaling pathway. Biosci Rep. 2019 Jul 30;39(7):BSR20182270. 10.1042/BSR20182270 PMC6667728 31262971

[13] Wu F, Chen Y, Zhu J. LHPP suppresses proliferation, migration, and invasion and promotes apoptosis in pancreatic cancer. Biosci Rep. 2020 Mar 27;40(3):BSR20194142. 10.1042/BSR20194142 PMC7103587 32186702

[14] Xia Z, et al. LHPP Inhibits the Viability, Migration, and Proliferation of PDAC Cells and Significantly Affects the Expression of SDC1 and S100p. Technol Cancer Res Treat. 2023 Jan-Dec;22:15330338231177807 10.1177/15330338231177807 PMC10278439 37321804

[15] Zheng J (2018). Down-regulation of LHPP in cervical cancer influences cell proliferation, metastasis and apoptosis by modulating AKT. Biochem. Biophys. Res. Commun..

[16] Sun W (2020). LHPP inhibits cell growth and migration and triggers autophagy in papillary thyroid cancer by regulating the AKT/AMPK/mTOR signaling pathway. Acta Biochim. Biophys. Sin. (Shanghai).

[17] Hou B (2020). Tumor suppressor LHPP regulates the proliferation of colorectal cancer cells via the PI3K/AKT pathway. Oncol. Rep..

[18] Hou B (2021). LHPP suppresses colorectal cancer cell migration and invasion in vitro and in vivo by inhibiting Smad3 phosphorylation in the TGF-β pathway. Cell Death Discov..

[19] Liao L (2020). LHPP inhibits hepatocellular carcinoma cell growth and metastasis. Cell Cycle.

[20] Zhang R (2016). Enhanced antitumor effect of combining TRAIL and MnSOD mediated by CEA-controlled oncolytic adenovirus in lung cancer. Cancer Gene Ther..

[21] Linder M, Liko D, Kancherla V, Piscuoglio S, Hall MN (2023). Colitis is associated with loss of the histidine phosphatase LHPP and upregulation of histidine phosphorylation in intestinal epithelial cells. Biomedicines.

[22] Zhu X (2023). Development and application of oncolytic viruses as the nemesis of tumor cells. Front. Microbiol..

[23] Chiocca EA, Rabkin SD (2014). Oncolytic viruses and their application to cancer immunotherapy. Cancer Immunol. Res..

[24] Ghasemi Darestani N (2023). Mesenchymal stem cell-released oncolytic virus: an innovative strategy for cancer treatment. Cell Commun. Signal..

[25] Malhotra J, Kim ES (2023). Oncolytic viruses and cancer immunotherapy. Curr. Oncol. Rep..

[26] Russell SJ, Peng KW, Bell JC (2012). Oncolytic virotherapy. Nat. Biotechnol..

[27] Lawler SE, Speranza MC, Cho CF, Chiocca EA (2017). Oncolytic viruses in cancer treatment: A review. JAMA Oncol..

[28] Liang M (2018). Oncorine, the world first oncolytic virus medicine and its update in China. Curr. Cancer Drug Targets.

[29] Wall LM, Baldwin-Medsker A (2017). Safe and effective standards of care: Supporting the administration of T-VEC for patients with advanced melanoma in the outpatient oncology setting. Clin. J. Oncol. Nurs..

[30] Frampton JE (2022). Teserpaturev/G47Δ: First approval. BioDrugs.

[31] Hindupur SK (2018). The protein histidine phosphatase LHPP is a tumour suppressor. Nature.

[32] Ma L (2022). Tumor suppressor LHPP suppresses cell proliferation and epithelial-mesenchymal transition in hepatocellular carcinoma cell lines. J. Physiol. Biochem..

[33] Wu F, Ma H, Wang X, Wei H, Zhang W, Zhang Y (2022). The histidine phosphatase LHPP: An emerging player in cancer. Cell Cycle.

[34] Zhu H, Song C, Li J, Liu Q, Liu M, Fu L (2023). LHPP suppresses proliferation, migration, and invasion in hepatocellular carcinoma and pancreatic cancer by inhibiting EGFR signaling pathway. Med. Oncol..

[35] Liu S (2022). As a novel tumor suppressor, LHPP promotes apoptosis by inhibiting the PI3K/AKT signaling pathway in oral squamous cell carcinoma. Int. J. Biol. Sci..

[36] Wang D, Li J, Li W (2022). LHPP suppresses gastric cancer progression via the PI3K/AKT/mTOR signaling pathway. J. Cancer.

[37] Thomas MA, Broughton RS, Goodrum FD, Ornelles DA (2009). E4orf1 limits the oncolytic potential of the E1B–55K deletion mutant adenovirus. J. Virol..

[38] Li X, Mao Q, Wang D, Zhang W, Xia H (2012). A fiber chimeric CRAd vector Ad5/11-D24 double-armed with TRAIL and arresten for enhanced glioblastoma therapy. Hum. Gene Ther..

[39] Wohlfahrt ME, Beard BC, Lieber A, Kiem HP (2007). A capsid-modified, conditionally replicating oncolytic adenovirus vector expressing TRAIL Leads to enhanced cancer cell killing in human glioblastoma models. Cancer Res..

[40] Kakiuchi Y, Kuroda S, Kanaya N, Kagawa S, Tazawa H, Fujiwara T (2023). Exosomes as a drug delivery tool for cancer therapy: A new era for existing drugs and oncolytic viruses. Expert Opin. Ther. Targets.

[41] Xiao B (2020). Doxorubicin hydrochloride enhanced antitumour effect of CEA-regulated oncolytic virotherapy in live cancer cells and a mouse model. J. Cell. Mol. Med..

[42] Huang Z (2023). Application of oncolytic virus in tumor therapy. J. Med. Virol..

[43] Fang L (2023). Oncolytic adenovirus-mediated expression of CCL5 and IL12 facilitates CA9-targeting CAR-T therapy against renal cell carcinoma. Pharmacol. Res..

[44] Li B, Chan HL, Chen P (2019). Immune checkpoint inhibitors: Basics and challenges. Curr. Med. Chem..

[45] Wolf Y, Anderson AC, Kuchroo VK (2020). TIM3 comes of age as an inhibitory receptor. Nat. Rev. Immunol..

[46] Vuchkovska A (2022). Siglec-5 is an inhibitory immune checkpoint molecule for human T cells. Immunology.

[47] Xiao B (2017). Combination of oncolytic adenovirus and luteolin exerts synergistic antitumor effects in colorectal cancer cells and a mouse model. Mol. Med. Rep..

