# Supplementary material for: Oncolytic adenovirus encoding LHPP exerts potent antitumor effect in lung cancer
Source: Sci Rep. 2024 Jun 7;14:13108. doi: 10.1038/s41598-024-63325-z (PMC11161505; doi:10.1038/s41598-024-63325-z)
Supplement: Supplementary file 2 — Supplementary Information. [file 41598_2024_63325_MOESM2_ESM.pdf]

Supplementary Figure Original images of Western blot

Figure 2b CAR

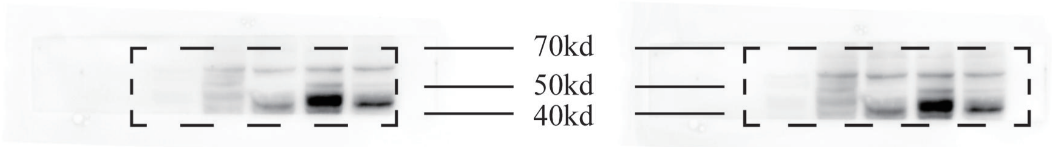

Exposure 5s Exposure 10s

Figure 2b GAPDH

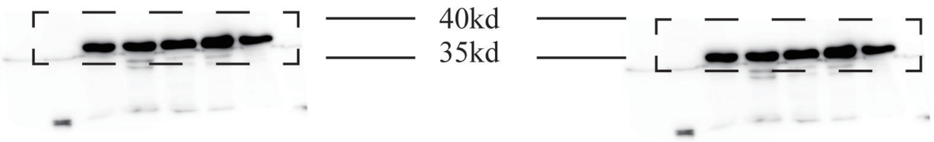

Exposure 1s Exposure 3s

Figure 2d LHPP

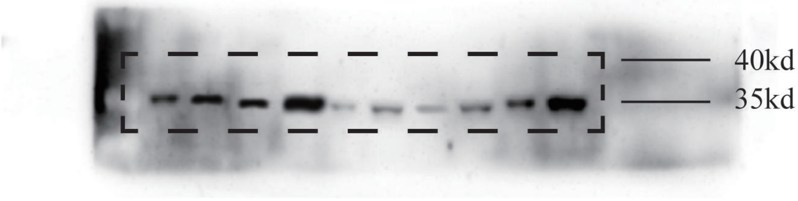

Exposure 40s

Figure 2d E1A

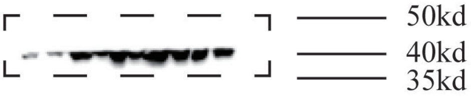

Exposure 30s

Figure 2d GAPDH

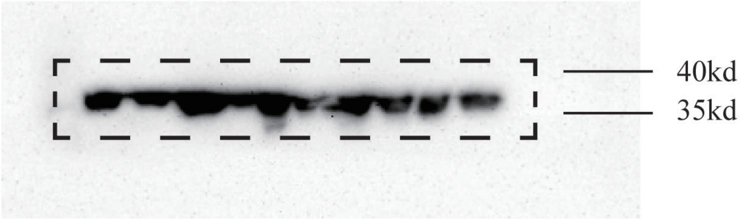

Exposure 10s

Figure 4c Caspase 3

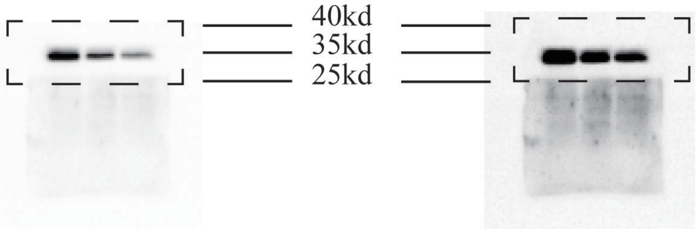

Exposure 60s Exposure 120s

Figure 4c Cleaved-Caspase 3

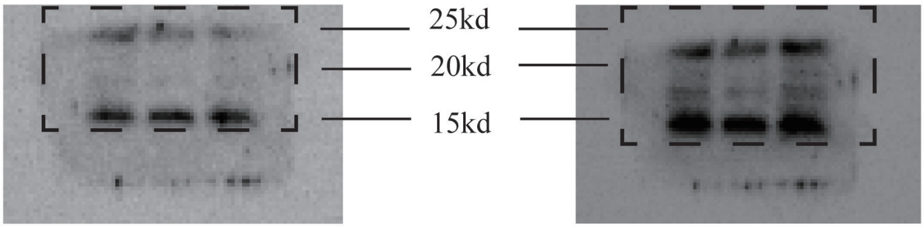

Exposure 40s Exposure 60s

Figure 4c BAX

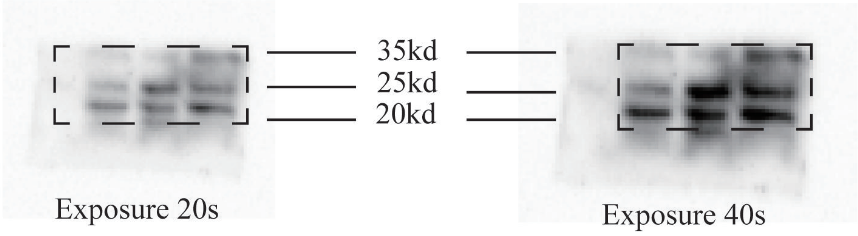

Figure 4c p62

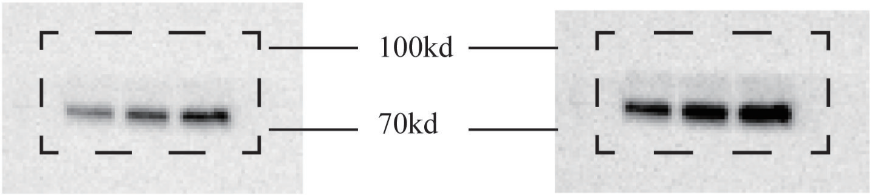

Exposure 20s   Exposure 40s

Figure 4c LC3A/LC3B

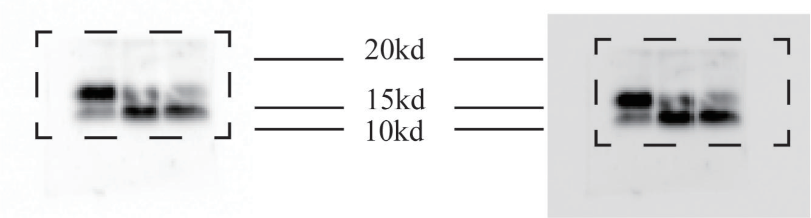

Exposure 30s   Exposure 60s

Figure 4c LHPP

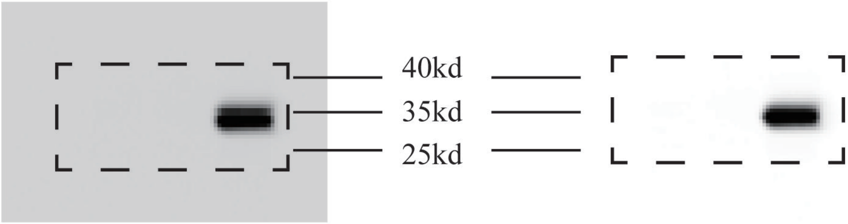

Exposure 20s   Exposure 30s

Figure 4c GAPDH

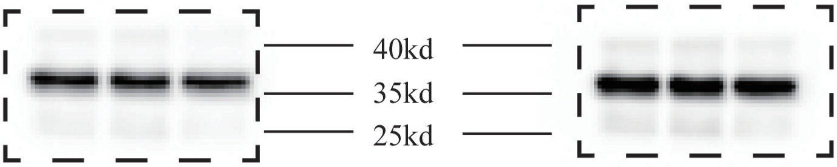

Exposure 20s   Exposure 30s

These original images from the blots used in our manuscript were scanned with a fully automated chemiluminescence image analyser, which was automatically exposed, and we didn't obtain multiple exposure images. Please understand.
